# Investigation of Methionine Metabolism in Coccolithophore by *In Situ* Light-Coupled Nuclear Magnetic Resonance Spectroscopy

**DOI:** 10.1021/acs.jpclett.5c01316

**Published:** 2025-06-03

**Authors:** Yi-Shan Wu, Li-Kang Chu, Tsyr-Yan Yu

**Affiliations:** † Institute of Atomic and Molecular Sciences, 38017 Academia Sinica, Taipei 106923, Taiwan; ‡ International Graduate Program of Molecular Science and Technology, 33561 National Taiwan University, No. 1, Section 4, Roosevelt Road, Daan District, Taipei 106923, Taiwan; § Molecular Science and Technology Program, Taiwan International Graduate Program (TIGP), 38017 Academia Sinica, No. 1, Section 4, Roosevelt Road, Daan District, Taipei 106923, Taiwan; ∥ Department of Chemistry, 34881 National Tsing Hua University, No. 101, Section 2, Kuang-Fu Road, Hsinchu 300044, Taiwan

## Abstract

Coccolithophores play critical roles in global carbon and sulfur cycles. They contribute to the carbon cycle through photosynthesis and calcification and the sulfur cycle by producing dimethylsulfoniopropionate (DMSP). Despite their ecological importance, the details and dynamics of methionine metabolism in coccolithophores are poorly understood. Here, we introduce an *in situ* light-coupled nuclear magnetic resonance (NMR) spectroscopy setup to monitor methionine metabolism directly in coccolithophore cultures under varying environmental conditions. Combining *in situ* light-coupled NMR spectroscopy and ^13^C magic angle spinning (MAS) spectroscopy, we observed that coccolithophores can take up methionine and convert it into 4-methylthio-2-oxobutyrate (MTOB), which is subsequently secreted into the culture medium, while DMSP was detected only intracellularly. Furthermore, environmental factors, such as elevated temperatures at 24.8 °C, which is 6.8 °C higher than the typical growth temperature for coccolithophores, and darkness, accelerated methionine consumption but reduced its incorporation into proteins and its conversion into MTOB, suggesting a shift toward alternative metabolic pathways under stress. In contrast, seawater acidification had minimal effects on the methionine metabolism. These findings provide new insights into how environmental conditions influence sulfur metabolism in coccolithophores, with potential consequences for their ecological functioning under future climate scenarios.

## Graphical abstract

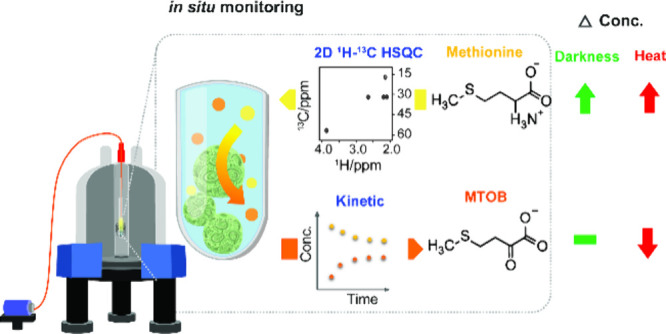

Phytoplankton are the ocean’s primary producers and play a crucial role in the biological carbon pump, fixing 30–50 billion metric tons of carbon each year.^1^ Among these phytoplankton, coccolithophores are unicellular microalgae known for their ability to produce minute calcium carbonate (CaCO_3_) flakes. They contribute approximately 20% of total organic carbon fixation^2,3^ and account for up to 50% of CaCO_3_ transported to marine sediments.^4–6^ Beyond their significance in the carbon cycle, coccolithophores also play an important role in the global sulfur cycle by producing dimethylsulfoniopropionate (DMSP).^7,8^ DMSP is not only a precursor to the climate-active gas dimethyl sulfide (DMS) but also serves as an important nutrient for marine microorganisms.^9^ Global annual emissions of DMS from the oceans are estimated to range from 13 to 37 teragrams of sulfur (Tg of S) per year.^10^ DMS is crucial for climate regulation by forming cloud condensation nuclei (CCN) via its oxidation products, such as dimethyl sulfoxide (DMSO) and sulfuric acid (H_2_SO_4_). These compounds contribute to cloud and aerosol formation, which, in turn, influence the Earth’s heat balance by altering aerosol–radiation interactions via scattering and absorbing solar radiation. As a result, DMS emissions can ultimately impact global temperatures.^11–13^ Additionally, DMSP functions as an osmolyte and a cryoprotectant, making it important for helping microorganisms survive in ice-cold environments.^14^ DMSP is also an essential source of carbon and sulfur in marine ecosystems and acts as a chemical signal to mediate marine microbial interactions, highlighting its importance in various environmental processes.^15,16^ Given the multifaceted roles of coccolithophores in these ecological systems, further research into their environmental impact is essential and cannot be overemphasized.

In the ocean, methionine can be incorporated in protein biosynthesis for microorganisms and serves as the precursor for the biosynthesis of DMSP, as illustrated in Scheme 1.^17–19^ It is widely believed that coccolithophores synthesize DMSP predominantly through a methionine transamination pathway, a hypothesis supported by the characterization of key enzymatic activities and identification of intermediate metabolites in marine bacteria and algae.^17,18^ However, alternative biosynthetic routes have also been proposed, including the methylation pathway and the decarboxylation pathway.^18^ The transamination reaction converts methionine to 4-methylthio-2-oxobutyrate (MTOB), which is then reduced to produce 4-methylthio-2-hydroxybutyrate (MTHB). DMSP is subsequently produced through methylation to form 4-dimethylsulfonio-2-hydroxybutyrate (DMSHB), followed by decarboxylation reactions. These intermediate compounds have been directly confirmed by mass spectrometry analysis of metabolites extracted from algae labeled with stable isotopes with the exception of MTOB. MTOB easily break down to 3-methylthiopropionate (MTP) in the standard methanol–chloroform–water extraction process, which prevents its direct quantification by mass spectrometry.^19^ As a result, MTOB is characterized and quantified indirectly. Because MTOB can be reduced to MTHB with NaBH_4_, quantification is achieved by comparing MTHB levels in paired extraction experiments, one with NaBH_4_ and the other with preneutralized NaBH_4_.^19^ While this conventional approach, combining stable isotope labeling and mass spectrometry, effectively elucidates the transamination pathway, extracting unstable trace chemical compounds secreted by coccolithophore cells in high-salt media remains challenging. This limitation offers only limited insights into the chemical compounds secreted and the kinetic parameters of DMSP biosynthesis. Consequently, there remains a gap in understanding how sulfur metabolism in coccolithophores influences their surrounding environment.

**Scheme 1:**
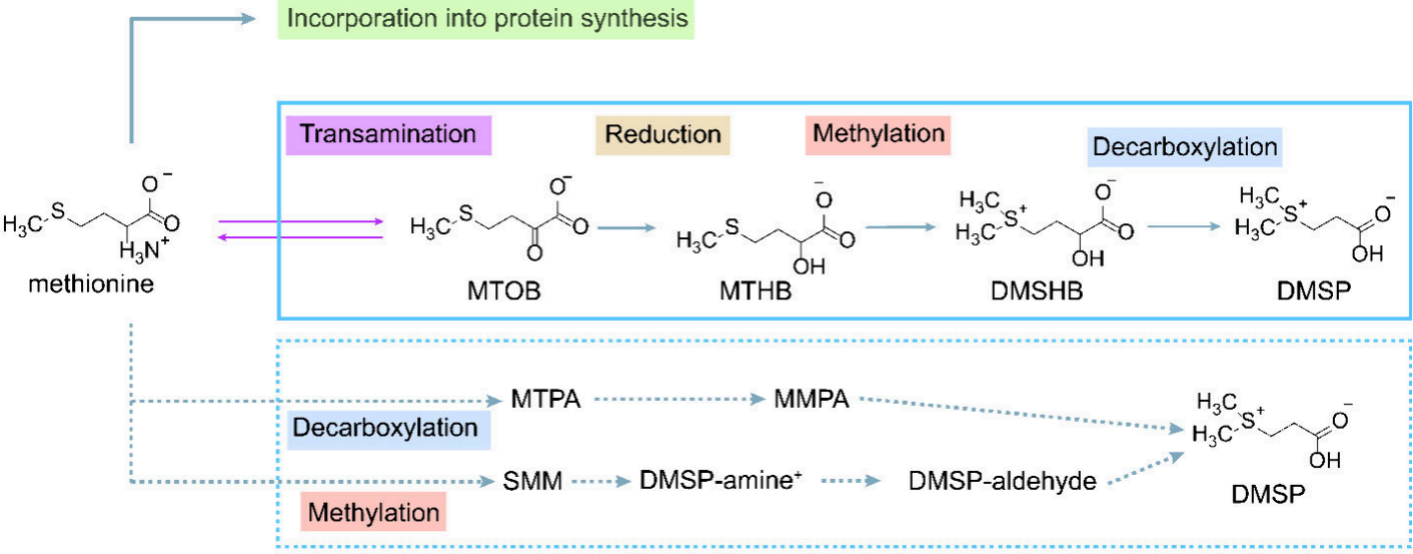
Proposed DMSP Biosynthetic Pathways in Coccolithophores^a^

a The transamination pathway, thought to be the hypothetical major pathway, is shown in the solid blue box, while the proposed decarboxylation and methylation pathways are shown in the dashed blue box.

Light-coupled nuclear magnetic resonance (NMR) spectroscopy has been successfully applied to investigate photochemical processes and light-sensitive biomolecules.^20–27^ In this study, we developed a light-coupled NMR detection system, as depicted in Figure 1, to enable *in situ* monitoring of coccolithophore cultures grown in ultraviolet (UV)-sterilized 5 mm Shigemi NMR tubes. Light illumination was provided by a halogen lamp (HL-2000-FHSA, Ocean Optics, Orlando, FL, U.S.A.), with light coupled to the NMR sample via a 1 mm diameter multimode optical fiber (QMMF-UVVIS-1000, OZ Optics, Ottawa, Ontario, Canada), which supports light transmission in the UV–visible spectral range. A ladder-shaped plunger was employed to ensure a uniform illumination of the sample. A Bruker NEO 850 MHz spectrometer, equipped with a 5 mm TCI (^1^H/^13^C/^15^N) CryoProbe with a *z*-axis gradient, was employed in all of the light-coupled NMR experiments. This light-coupled setup mimics the natural growth conditions of coccolithophores by providing light illumination. The Emiliania huxleyi RCC1216 culture was initially grown in a Nunc EasYFlask-25 T cell culture flask containing 30 mL of K/2 medium at 18 °C. The recipes for preparing the K/2 medium are documented in –,^28^ and the typical growth curve is shown in . For the *in situ* NMR experiment, a typical sample consisted of 330 μL of coccolithophore culture with a cell density ranging from 1.5 to 2 × 10^6^ cells/mL, mixed with 0.5 μL of 37 mM [U-^13^C]-labeled methionine and 2.3 μL of 80 mM 3-(trimethylsilyl)­propionic-2,2,3,3-*d*_4_ acid (TSP-*d*_4_), followed by the addition of D_2_O to achieve a final concentration of 10% (v/v). After the mixture was transferred to an UV-sterilized 5 mm Shigemi NMR tube, the coccolithophore cells rapidly settled at the bottom, allowing us to effectively monitor the culture medium. Unlike conventional studies, which typically focus on analyzing methionine-derived metabolites extracted from coccolithophore cells, our method enables the analysis of metabolites secreted by the coccolithophores, providing a direct observation of their influence on the surrounding environment. We recorded two-dimensional (2D) ^13^C-^1^H heteronuclear single quantum correlation (HSQC) spectra to investigate the metabolism of [U-^13^C]-labeled methionine *in situ*. All *in situ* 2D ^13^C-^1^H HSQC spectra were acquired using the standard Bruker pulse sequence hsqcetgpsisp2.2, with the spectral widths set to 10.138 and 140 ppm for the direct (^1^H) dimension and indirect (^13^C) dimension, respectively. Each spectrum was acquired with 512 complex points in the direct dimension and 50 complex points in the indirect dimension. The number of scans for each experiment was set to be 136. Due to the high salt content in the coccolithophore culture medium, the ^1^H 90° pulse width at a power level of −12 dB was determined to be 14 μs. It is worth noting that the acquisition time for each spectrum was 6 h, which provided sufficient spectral sensitivity and, more importantly, allowed the secreted compounds to diffuse into the detection zone. As shown in , the spectrum of the coccolithophore culture recorded immediately after thorough mixing is nearly identical with that recorded before mixing. These spectra serve as fingerprints for chemical compounds, allowing for the identification of intermediate metabolites derived from [U-^13^C]-labeled methionine. The NMR spectra of standard compounds that may be derived from methionine, such as MTOB, MTHB, and DMSP, are shown in , with their chemical shifts summarized in . Although DMSHB is not commercially available, some of its ^1^H chemical shifts have been previously reported and are summarized in .

**Figure 1:**
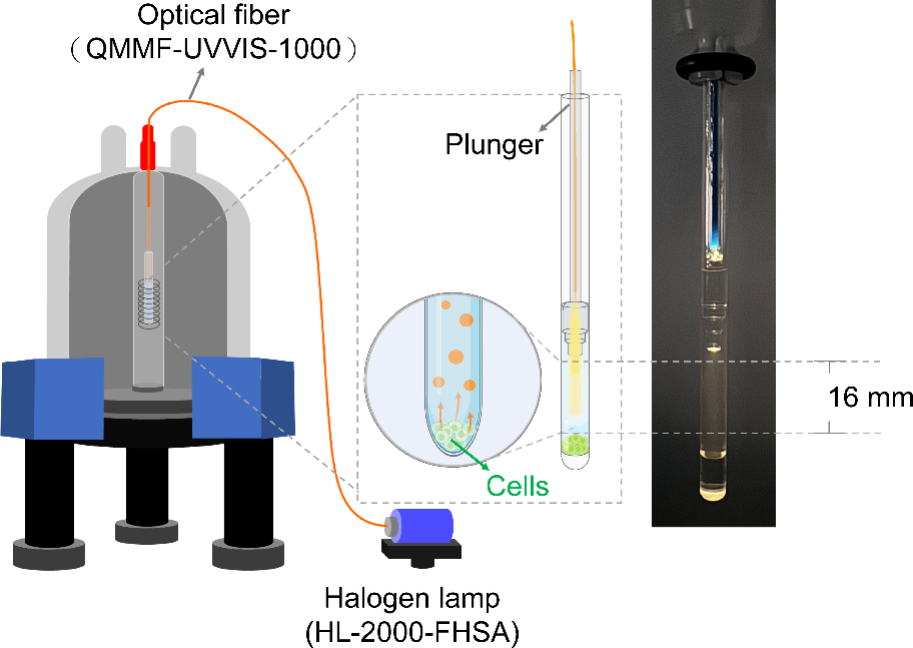
Light-coupled NMR detection system for the *in situ* monitoring of coccolithophore cultures.

During the first 6 h after the addition of [U-^13^C]-labeled methionine, we detected only [U-^13^C]-labeled methionine in the culture medium, with no intermediate compounds present, as shown in Figure 2a. However, between 30 and 36 h after the addition of [U-^13^C]-labeled methionine, we detected a significant accumulation of [U-^13^C]-labeled MTOB but no other compounds derived from [U-^13^C]-labeled methionine in the culture medium, as shown in Figure 2b. This result indicates that a substantial amount of methionine was converted to MTOB and secreted outside of the cells. Notably, although DMSP can be detected in coccolithophore cell extracts using liquid chromatography–mass (LC–MS) spectrometry, as shown in , it was not detectable in the culture medium by NMR spectroscopy.^29^

**Figure 2:**
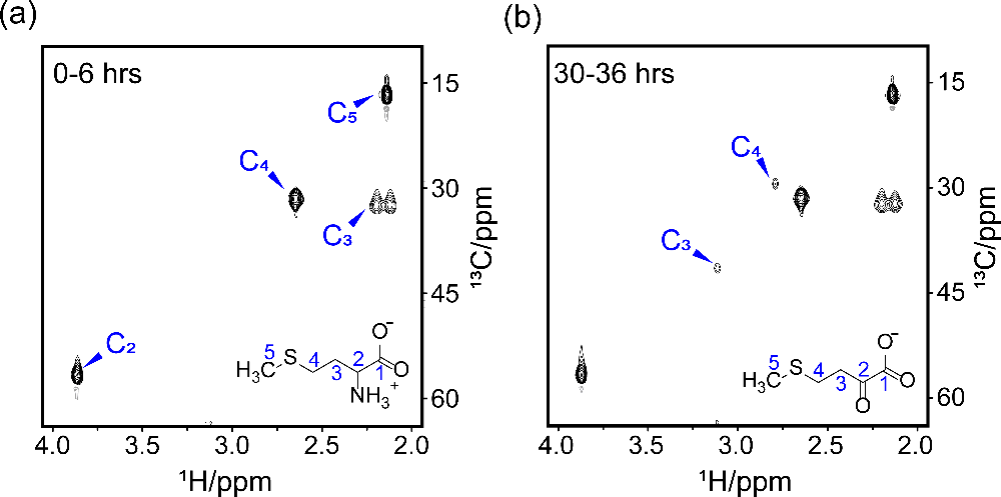
*In situ* NMR spectroscopy used to monitor methionine metabolism in a coccolithophore culture. (a) 2D ^13^C-^1^H HSQC NMR spectrum recorded during the first 6 h after the addition of 50 μM [U-^13^C]-labeled methionine. (b) Spectrum recorded from 30 to 36 h after the addition of [U-^13^C]-labeled methionine.

Coccolithophore growth is known to be influenced by environmental factors, such as the temperature, light–dark cycles, and seawater acidity.^30–34^ To explore whether these factors impact methionine metabolism, we employed our light-coupled NMR detection setup in combination with ^13^C magic angle spinning (MAS) NMR spectroscopy. The light-coupled NMR detection primarily provides information on metabolites present in the growth medium, reflecting extracellular or environmental changes, while ^13^C MAS NMR reveals intracellular metabolic activity within the coccolithophore cells. This study aimed to monitor the kinetics of [U-^13^C]-labeled methionine consumption, along with the production and secretion of its derivatives, by coccolithophore cells under stress conditions and to assess their impact on methionine incorporation into protein synthesis. Given that both the frequency and duration of marine heat waves have increased in recent decades,^35,36^ we investigated how elevated seawater temperatures associated with heat waves affect methionine metabolism. Here, we compared methionine consumption and MTOB production in light-grown coccolithophores cultured at 18 and 24.8 °C, with the latter representing the record-high sea surface temperature observed in 2022.^34,36^ In addition to the temperature, we also investigated the impact of light illumination conditions by comparing coccolithophores grown under continuous light and complete darkness, allowing us to evaluate how light availability influences methionine utilization and metabolite secretion. Our results showed that the consumption of methionine in the culture medium at the elevated temperature was significantly higher than that at 18 °C, especially during the first 24 h following the addition of [U-^13^C]-labeled methionine. We observed approximately 18% of the methionine in the culture medium consumed by the coccolithophore culture grown at 24.8 °C during the first 24 h compared to less than 5% at 18 °C, as shown in Figure 3a. In contrast, the MTOB concentration in the medium of the coccolithophore culture grown at 18 °C increased steadily, while the MTOB concentration in the culture grown at 24.8 °C plateaued after 18 h, as shown in Figure 3b.^37^ Additionally, a slight increase in methionine consumption was observed in dark-grown coccolithophores,^38^ but MTOB production remained largely unaffected by light conditions. These results suggest that not all consumed methionine is converted into MTOB under dark or stress conditions but likely redirected into alternative metabolic pathways.

**Figure 3:**
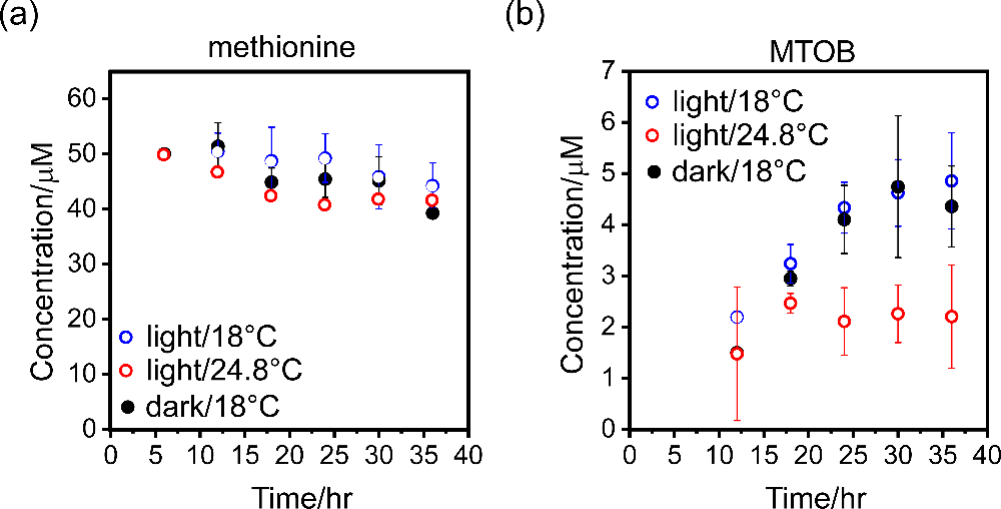
Methionine consumption and MTOB production in coccolithophore culture over time following the addition of 50 μM [U-^13^C]-labeled methionine. Data were collected at 18 °C under light, 24.8 °C under light, and 18 °C under dark conditions. Concentrations of (a) methionine and (b) MTOB were monitored over time by using 2D ^13^C-^1^H HSQC NMR spectroscopy.

Complementing the *in situ* light-coupled NMR used to monitor the concentrations of [U-^13^C]-labeled methionine and its derivatives in the coccolithophore growth medium, ^13^C MAS NMR spectra were recorded from coccolithophore cell pellets to investigate the distribution of ^13^C-labeled species within the coccolithophore cells grown under four different conditions. All ^13^C MAS NMR spectra were acquired on a Bruker wide-bore 11.7 T Avance III 500 MHz spectrometer equipped with a 3.2 mm triple-resonance MAS probe. The sample spinning rate was set to 8 kHz, and the recycle delay was set to 5 s. Hahn echo spectra were recorded with ^13^C radio-frequency pulses at a field strength of 50 kHz. The spectral width was 795 ppm with a transmitter offset of −150.996 ppm. Final spectra were obtained by accumulating 10 000 scans. To prepare the samples for ^13^C MAS NMR experiments, each coccolithophore culture of 300 mL was derived from a common seed culture and initially grown at 18 °C under continuous light until reaching the cell density of 2.0 × 10^6^ cells/mL. Of the four cultures, one was used as a reference and received no [U-^13^C]-labeled methionine. The remaining three cultures were each supplemented with 258.6 μL of 58 mM [U-^13^C]-labeled methionine to achieve a final concentration of 50 μM. These cultures were subsequently incubated for an additional 24 h under the various conditions: 18 °C under light, 18 °C in darkness, and 24.8 °C under light, respectively. Coccolithophore cells were harvested by centrifugation at 3000*g* for 15 min, lyophilized, and packed into 3.2 mm MAS rotors. The ^13^C MAS spectra were normalized using the resonance peak associated with calcite, which appears at 168.6 ppm. The broad resonance peaks ranging from 170 to 179 ppm are associated with carbonyl carbons in protein or peptides. As shown in Figure 4a, under light-grown conditions at 18 °C, coccolithophores incorporated additional [U-^13^C]-labeled methionine into protein synthesis, resulting in greater intensity for the peak ranging from 170 to 179 ppm. In contrast, cells incubated either in darkness at 18 °C, shown in Figure 4b, or under elevated temperature at 24.8 °C with light, shown in Figure 4c, did not show a comparable increase in this region. As discussed earlier, coccolithophore cultures grown under the conditions of darkness at 18 °C or at an elevated temperature (24.8 °C) with light exhibited increased methionine consumption compared to the cultures grown at 18 °C under light. However, despite this increased consumption, the ^13^C MAS NMR spectra did not show a corresponding increase in the carbonyl resonance region (170–179 ppm), which typically reflects incorporation of methionine into protein synthesis. Taken together, these findings suggest that excess methionine was neither converted into MTOB nor significantly utilized for protein biosynthesis in coccolithophores but was instead channeled into alternative metabolic pathway(s). A comparison of the ^13^C MAS NMR spectra in the aliphatic carbon region revealed new peaks at 41.09 and 16.49 ppm in samples from coccolithophores incubated in darkness or at elevated temperatures, shown in . These peaks correspond to C1 and methylmercapto carbons of 3-methylthiopropylamine (MTPA), respectively, suggesting that methionine may have potentially been diverted into the decarboxylation pathway.

**Figure 4:**
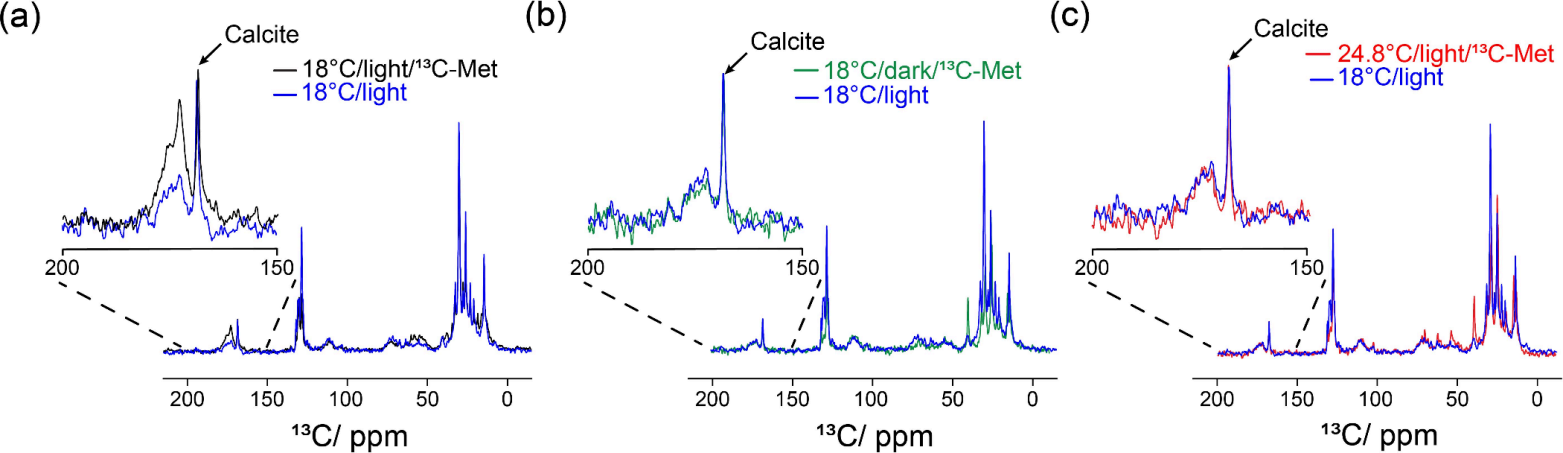
^13^C MAS spectra of coccolithophore pellets collected after 24 h of incubation under various conditions following the addition of 50 μM [U-^13^C]-labeled methionine. A reference spectrum was obtained from coccolithophores grown at 18 °C under light conditions without the addition of [U-^13^C]-labeled methionine. Overlay of the reference spectrum and the ^13^C MAS spectrum of the coccolithophore pellet collected after 24 h of incubation at (a) 18 °C under light, (b) 18 °C in darkness, and (c) 24.8 °C under light, following the addition of 50 μM [U-^13^C]-labeled methionine.

Finally, the CO_2_ concentration in the atmosphere has increased in recent decades, and seawater acidification has become a significant concern, stimulating growing interest in understanding how acidity affects coccolithophore biology.^39^ To investigate methionine metabolism under different pH conditions, NMR experiments were performed with coccolithophore cultures maintained in the three media prepared at pH 8.18, 8.0, and 7.6, respectively.^40^ Each coccolithophore culture was initiated by adding a seed culture to 30 mL of culture medium, which was then maintained for 12 days before introducing [U-^13^C]-labeled methionine. Our results indicate that the variation in seawater pH, ranging from 8.18 to 7.6, had no significant effect on methionine consumption or the secretion of MTOB from the cells.^41^ This observation is supported by the 2D ^13^C-^1^H HSQC spectra, shown in Figure 5, which reveal nearly identical profiles for the cultures at the three pH values, collected 24 h after the addition of [U-^13^C]-labeled methionine.

**Figure 5:**
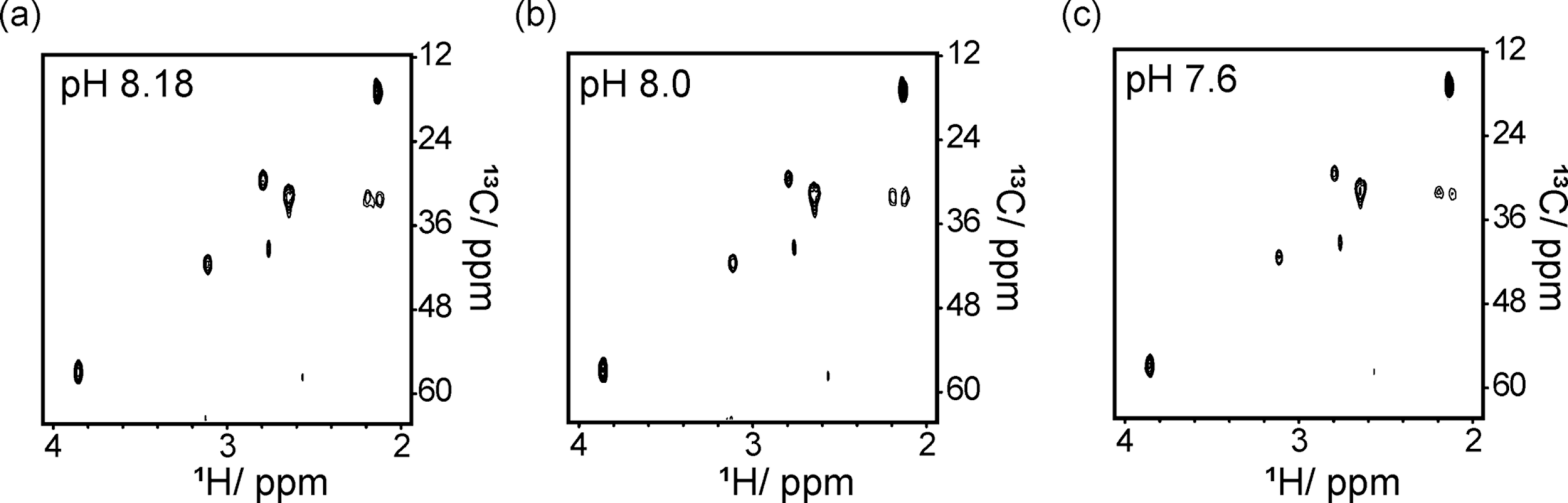
2D ^13^C-^1^H HSQC spectra of coccolithophores grown in culture media at pH values of (a) 8.18, (b) 8.0, and (c) 7.6. The spectra were recorded 24 h after the addition of [U-^13^C]-labeled methionine.

In summary, our research provides an *in situ* analysis of methionine metabolism in coccolithophores under various environmental conditions, offering new insights into their roles in the oceanic sulfur cycle. Specifically, we demonstrate that, under stress conditions, such as in darkness or at elevated temperatures, coccolithophores do not channel all consumed methionine into MTOB biosynthesis via the transamination pathway nor do they incorporate methionine into protein synthesis. Importantly, the *in situ* light-coupled NMR approach employed in this study enables the direct detection of MTOB, overcoming a key limitation of conventional methods that rely on mass spectrometry combined with isotope labeling. The findings are particularly relevant for understanding how climate-change-related stress factors, such as rising temperatures and ocean acidification, affect phytoplankton metabolism, which could have broader implications for marine ecosystems and global climate regulation.

## Supplementary Material





## Notes

Materials and experimental methods, scanning electron microscopy (SEM), flow cytometry, and LC–MS characterizations, and 2D ^13^C-^1^H HSQC spectra of compounds involved in this work (PDF)
Transparent Peer Review report available (PDF)
