# Supplementary material for: Investigation of Methionine Metabolism in Coccolithophore by In Situ Light-Coupled Nuclear Magnetic Resonance Spectroscopy
Source: J Phys Chem Lett. 2025 Jun 3;16(23):5800–5. doi: 10.1021/acs.jpclett.5c01316 (PMC12169660; doi:10.1021/acs.jpclett.5c01316)
Supplement: Supplementary file 1 [file jz5c01316_si_001.pdf]

Supporting information

Investigation of methionine metabolism in coccolithophore by *in situ* light-coupled NMR spectroscopy

Yi-Shan Wu,[a,b,c], Li-Kang Chu,[d] Tsyr-Yan Yu,[a,b,c*]*

[a]Institute of Atomic and Molecular Sciences, Academia Sinica, Taipei 106923, Taiwan

[b]International Graduate Program of Molecular Science and Technology, National Taiwan University No. 1, Sec. 4, Roosevelt Rd., Daan Dist., Taipei, 106923, Taiwan

[c]Molecular Science and Technology Program, Taiwan International Graduate Program (TIGP), Academia Sinica No. 1, Sec. 4, Roosevelt Rd., Daan Dist., Taipei, 106923, Taiwan

[d]Department of Chemistry, National Tsing Hua University, No. 101, Sec. 2, Kuang-Fu Road, Hsinchu, 300044, Taiwan

Corresponding Authors

*Li-Kang Chu

Tel: +886-3-5715131 /E-mail: lkchu@mx.nthu.edu.tw

*Tsyr-Yan Yu

Tel: +886-2-2366-8210/E-mail: dharmanmr@gate.sinica.edu.tw

Table of Contents

Growth and Monitoring of *Emiliania huxleyi* (RCC1216) Culture.**Error! Bookmark not defined.**

Detection of DMSP in Coccolithophore Extracts……………………………………….............2

Table S1. Recipe for modified K/2 medium (1L)..5

Table S2. Recipe for ASW salt dissolved in 994 mL ddH$_2$O………………………..................6

Table S3. Recipe for f/2 vitamin solution………………………………………………. …….6

Table S4. Recipe for trace metal solution…………………………………………………...7

Table S5. Paired ^{13}C/^{1}H chemical shift values of compounds involved in methionine metabolism, as obtained from the cross peaks in the 2D [^{13}C,^{1}H] HSQC spectra……………..7

Figure S1. The growth curve of *E. huxleyi* (RCC1216) cultured in modified K/2 medium.......8

Figure S2. Overlay of 1D [^{13}C,^{1}H] HSQC spectra recorded from a coccolithophore sample after 24 h of static incubation and immediately after thorough mixing to ensure sample homogeneity……………………………………………………………………9

Figure S3 The 2D [^{13}C,^{1}H] HSQC spectra of the standard compounds involved in the methionine metabolism pathway…………………………………………………………...10

Figure S4. Flow cytometry dot plot and histogram of red fluorescence emitted by chlorophyll a at an emission wavelength of 690 nm……………………………………………………11

Figure S5. Scanning electron microscope (SEM) images of the 15-day *E. huxleyi* (RCC1216) cells………………………………………………………….................................12

Figure S6. LC-MS chromatogram and mass spectrum of the 12-day *E. huxleyi* culture extract. ………………………………………………………………………….…………13

Figure S7. ^{13}C MAS spectra (0-50 ppm, aliphatic region) of coccolithophore pellets incubated for 24 h under different conditions following the addition of 50 µM [U-^{13}C] methionine.……………………………………………………………………………14

Growth and Monitoring of *Emiliania huxleyi* (RCC1216) Culture

E. huxleyi (RCC1216) was cultured in Nunc™ EasYFlask™-25 T cell culture flasks containing 30 mL of sterile-filtered K/2 medium and incubated at 18°C under continuous light with an irradiance of 120 μmol m^{-2} s^{-1} photon flux density in a growth chamber. The cell density was monitored using a flow cytometer (B75408 CytoFlex S; Beckman, Brea, USA) with an excitation wavelength of 488 nm. The cell population and viability of the coccolithophore cultures, with and without the addition of [U-^{13}C] labeled methionine, were assessed using flow cytometry dot plots (Figure S4), with forward scatter intensity plotted on the x-axis and side scatter intensity on the y-axis, along with the detection of red fluorescence from chlorophyll a at an emission wavelength of 690 nm. Scanning electron microscopy (Phenom Pharos G2 Desktop FEG-SEM, Thermo Fisher Scientific, Waltham, USA) was used to further characterize the cultured coccolithophore. For example, the 15-day *E. huxleyi* (RCC1216) culture was harvested by gravity filtration using Whatman GF/C (0.3 μm) filter paper and subsequently analyzed by SEM, as shown in Figure S5.

Detection of DMSP in Coccolithophore Extracts

Prior to the extraction experiment, 50 mL of *E. huxleyi* culture were grown under continuous light irradiation at 18°C for 12 days, until the cell density reached 2×10^6

cells per mL. The cells were harvested using gravity filtration on a Whatman GF/C (0.3 μm) filter and transferred into glass sample vials containing 1 mL of methanol, then stored at −20 °C. After 4 days of static extraction, 100 μL of the extracts were diluted with 900 μL of a water/acetonitrile (10:90, v/v) mixture and centrifuged at 10,000 × g for 5 minutes to remove cell pellets.[29] The resulting supernatant was analyzed using liquid chromatography-electrospray ionization mass spectrometry (LC-ESI-MS) with an LTQ Orbitrap XL ETD mass spectrometer (Thermo Fisher Scientific, San Jose, CA) coupled to a Waters Acquity UPLC system (Waters, Milford, MA). Chromatographic separation was performed on a BEH Amide column (1 mm I.D. × 100 mm, 1.7 μm, 130 Å, Waters), with 0.1% formic acid in water as mobile phase A and 0.1% formic acid in 100% acetonitrile as mobile phase B. The flow rate was set to 50 μL/min. A gradient elution was applied, starting at 70% buffer B and decreasing to 53% buffer B over 3 to 40 minutes. DMSP in the cell extracts was identified using LC-MS in single ion monitoring (SIM) mode at a mass-to-charge (m/z) ratio of 135.045, and confirmation was achieved through the electron ionization (EI) chromatogram, as depicted in Figure S6.

Table S1. Recipe for modified K/2 medium (1L).

Component	Amount (mL)
Artificial seawater salts (ASW)[#]	994
$NaNO_3$ (576 mM)	0.5
KH_2PO_4 (36 mM)	0.5
(Na)Fe · EDTA (5.86 mM)	0.5
Trace metal[&]	0.5
NH_4Cl (10 mM)	0.5
f/2 vitamin stock[$]	0.1
ddH_2O	3.4

[#]The recipe for ASW salts dissolved in 994 mL ddH_2O is listed in Table S2.

[$]The recipe for f/2 vitamin solution is listed in Table S3.

[&]The recipe for trace metal solution is listed in Table S4.

Adjust the pH of the medium to 8.18 using NaOH. Afterward, sterilize the modified medium by filtration and autoclaving.

Table S2. Recipe for ASW salt dissolved in 994 mL ddH_2O.*

Compounds	Amount (mg)
NaCl	24,600
KCl	750
$NaHCO_3$	210
H_3BO_3	36
KBr	116
$MgCl_2 \cdot 6H_2O$	4,070
$CaCl_2 \cdot 2H_2O$	1,470
$MgSO_4 \cdot 7H_2O$	6,180
NaF	2.90

* After adding other components of modified K/2 medium, the final volume is 1000 mL.

Table S3. Recipe for f/2 vitamin solution.

Compound	Stock solution concentration (mM)
Thiamine HCl	740
Biotin	5.00
Vitamin B12	1.84

Table S4. Recipe for trace metal solution.

Compound	Stock solution (mM)
$CuSO_4.5H_2O$	100
$Na_2MoO_4.2H_2O$	300
$ZnSO_4.7H_2O$	800
$CoSO_4.7H_2O$	500
$MnCl_2.4H_2O$	900
H_2SeO_3	100
$NiCl_2.6H_2O$	63

Table S5. Paired $^{13}C/^{1}H$ chemical shift values of compounds involved in methionine metabolism, as obtained from the cross peaks in the 2D [^{13}C,^{1}H] HSQC spectra.

Compounds	Carbon number	^{13}C/ppm	^{1}H/ppm
L-methionine	C5	16.5	2.145
	C4	31.5	2.658
	C3	32.6	2.127/2.208
	C2	56.8	3.879
MTOB<br>4-methylthio-2-oxobutyrate	C5	16.8	2.14
	C4	29.4	2.8
	C3	41.3	3.131
MTHB<br>4-methylthio-2-hydroxybutyrate	C5	16.5	2.131
	C4	31.6	2.613
	C3	36.1	1.933/2.037
	C2	73.9	4.181
DMSP	C4	27.6	2.947
	C3	33.9	2.757
	C2	43.1	3.469
DMSHB<br>4-dimethylsulfonio-2-hydroxybutyrate	C6/C5	-	2.91
	C4	-	3.34
	C3	-	3.44
	C	-	-

3-(Trimethylsilyl)propionic-2,2,3,3-d_4 acid sodium salt was used as the chemical shift reference.

$ DMSHB is not commercially available. Its ^{1}H chemical shift values were obtained from reference 18, which used the pyrazine resonance at 8.64 ppm as the chemical shift reference.

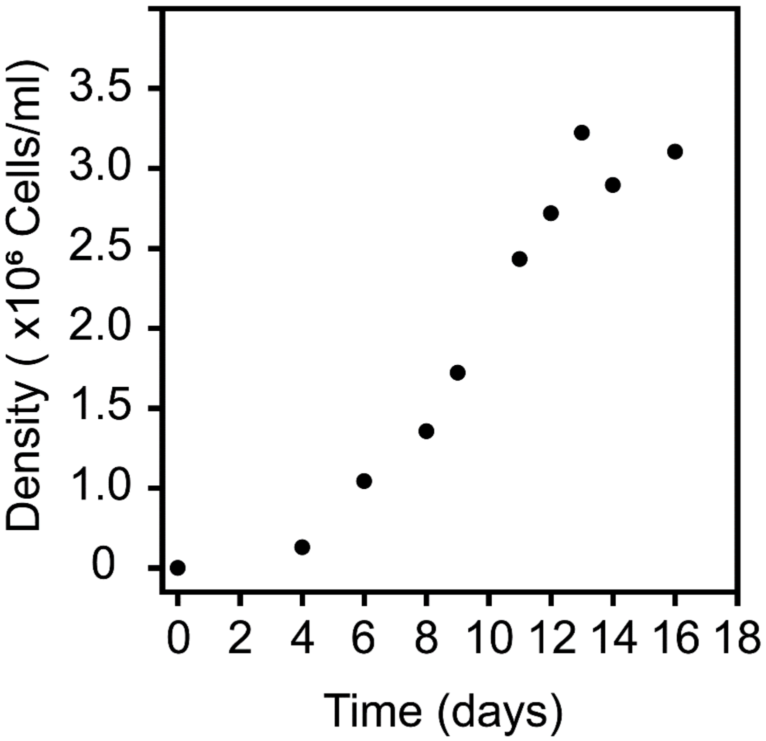

Figure S1. The growth curve of *E. huxleyi* (RCC1216) cultured in modified K/2 medium.

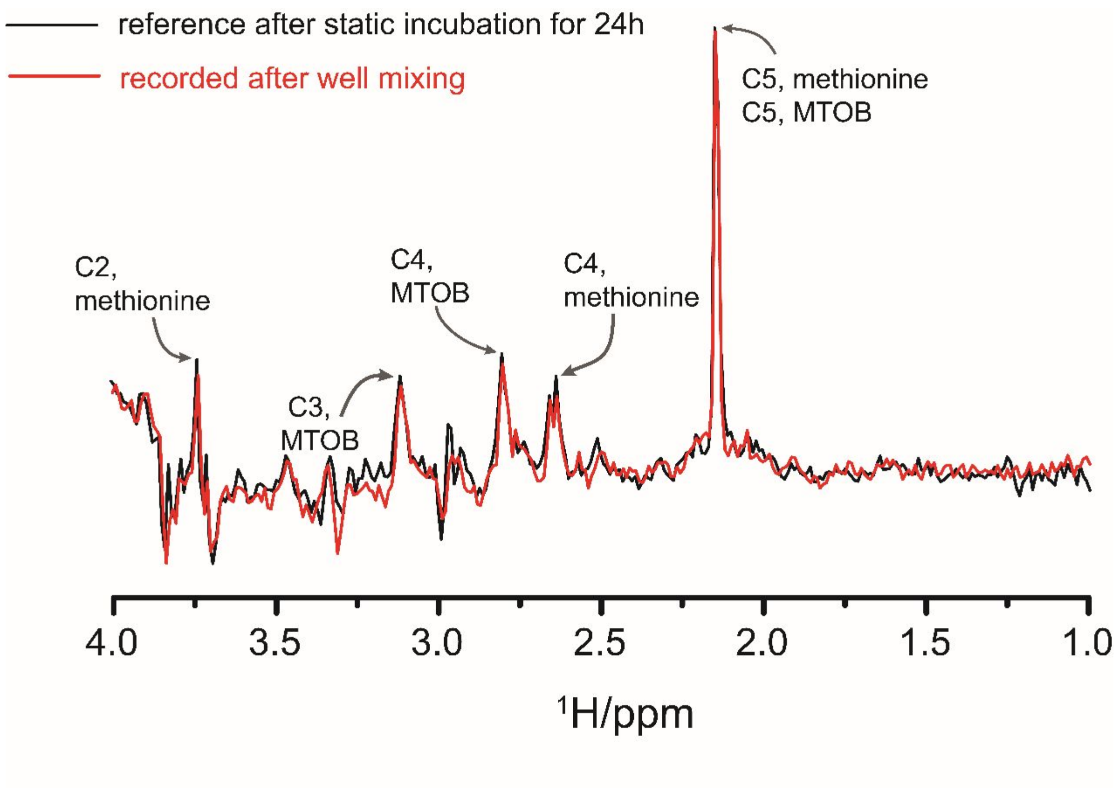

Figure S2. Overlay of 1D [^{13}C,^{1}H] HSQC spectra recorded from a coccolithophore sample after 24 h of static incubation and immediately after thorough mixing to ensure sample homogeneity. The coccolithophore culture was grown to a density of 2×10^6 cells per mL and supplemented with 0.5 µL of 37 mM [U-^{13}C] labeled methionine and 37 µL of D_2O prior to static incubation at 18°C for 24 h. After recording the reference 1D HSQC spectrum, the sample was immediately mixed thoroughly, and a second spectrum was recorded. The experiments were performed on a Bruker AVIII 600 MHz spectrometer, equipped with a 5 mm TXI (^{1}H/^{13}C/^{15}N) CryoProbe with a z-axis gradient. The 1D [^{13}C,^{1}H] HSQC spectra were acquired using the standard Bruker pulse sequence hsqcetgpsisp2.2, with a spectral width of 10 ppm for the direct (^{1}H) dimension. The spectrum was acquired with 1024 complex points in the direct dimension, and 800 scans were collected. The ^{1}H 90° pulse width at a power level of -10.17 dB was determined to be 15.26 µs.

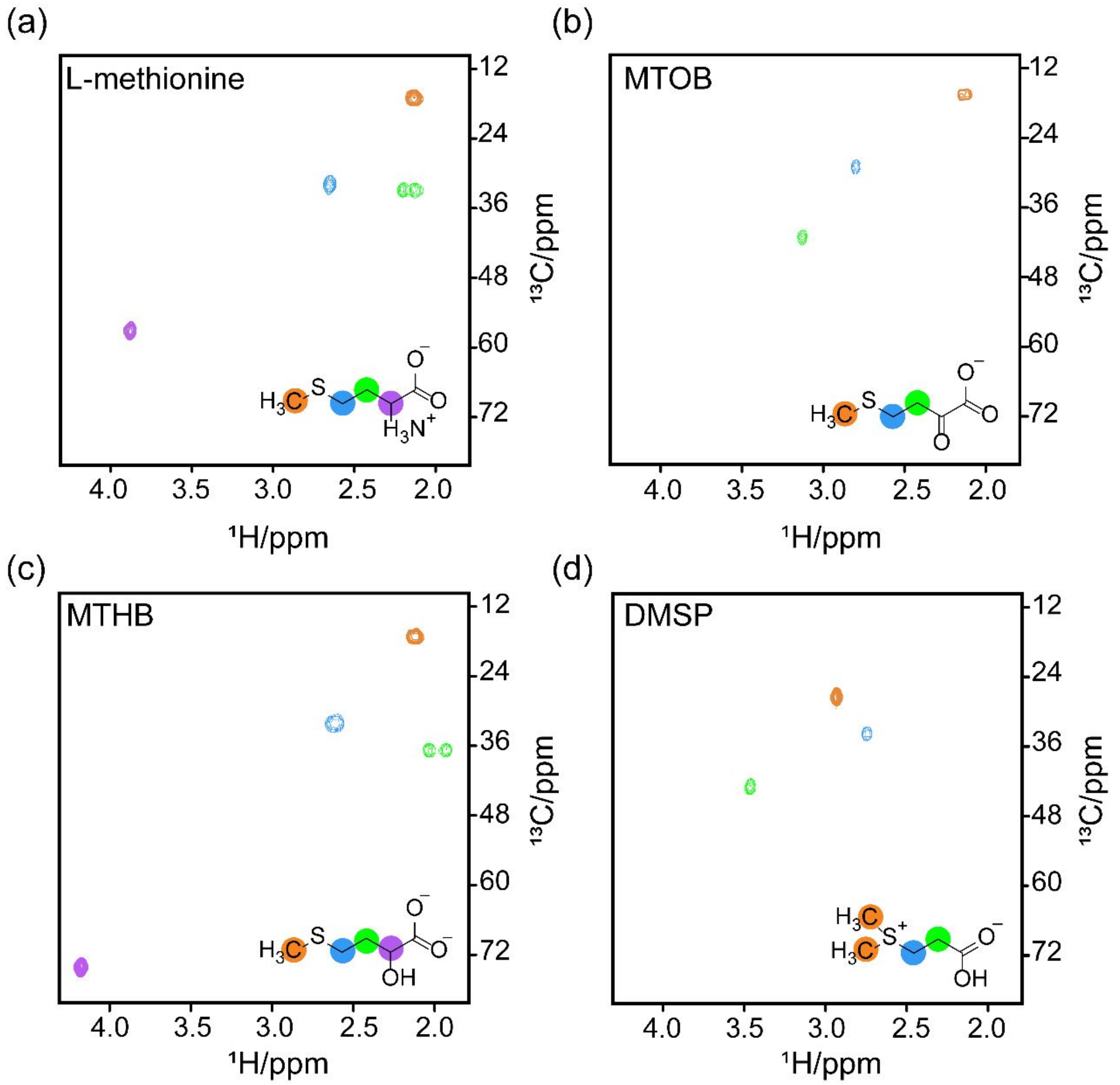

Figure S3. The 2D [^{13}C,^{1}H] HSQC spectra of the standard compounds, involved in the methionine metabolism pathway including methionine (a), MTOB (b), MTHB (c), and DMSP (d). Each compound was dissolved in modified K/2 medium at a concentration of 1.5 mM. Each spectrum was acquired using the standard Bruker pulse sequence hsqcetgpsisp.2, with the spectral widths set to 14 ppm and 200 ppm for the direct (^{1}H) dimension and indirect (^{13}C) dimension, respectively. Each spectrum was acquired with 512 complex points in the direct dimension and 64 complex points in the indirect dimension. The number of scans for each experiment was set to be 12. Due to the high salt content in the K/2 medium, the ^{1}H 90° pulse width at a power level of –11.87 dB was determined to be 17 μs.

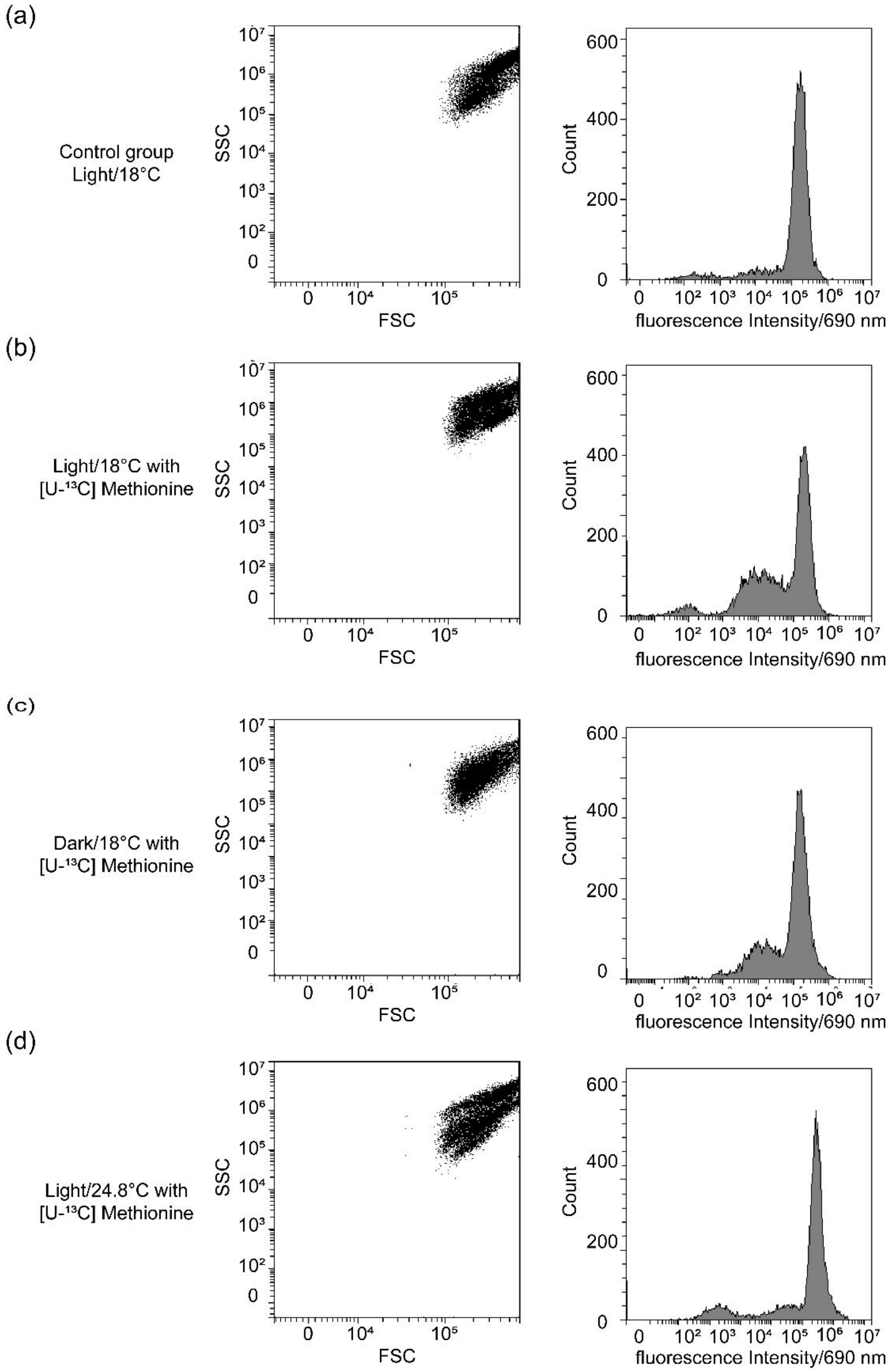

Figure S4. Flow cytometry dot plot and histogram of red fluorescence emitted by chlorophyll a at an emission wavelength of 690 nm for coccolithophore cultures grown to saturation under continuous illumination at 18 °C (a), and after 24 h of incubation with 50 µM [U-^{13}C] methionine under the following conditions: 18 °C with light (b), 18 °C in darkness (c), and 24.8 °C with light (d).

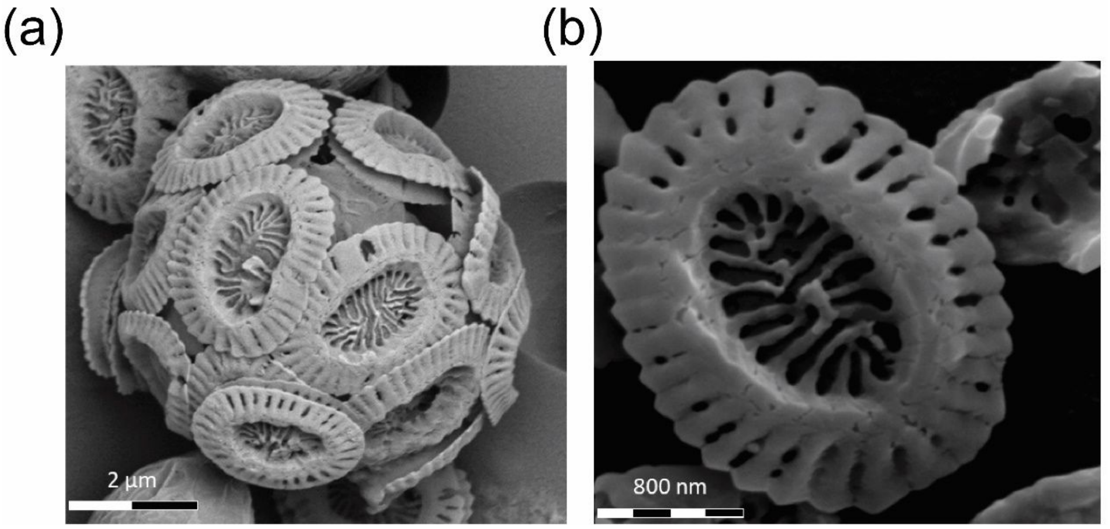

Figure S5. Scanning electron microscope (SEM) images of the 15-day *E. huxleyi* (RCC1216) cells. (a) Coccolith-bearing cell and coccosphere, and (b) coccolith, calcium carbonate plates formed by the coccolithophores.

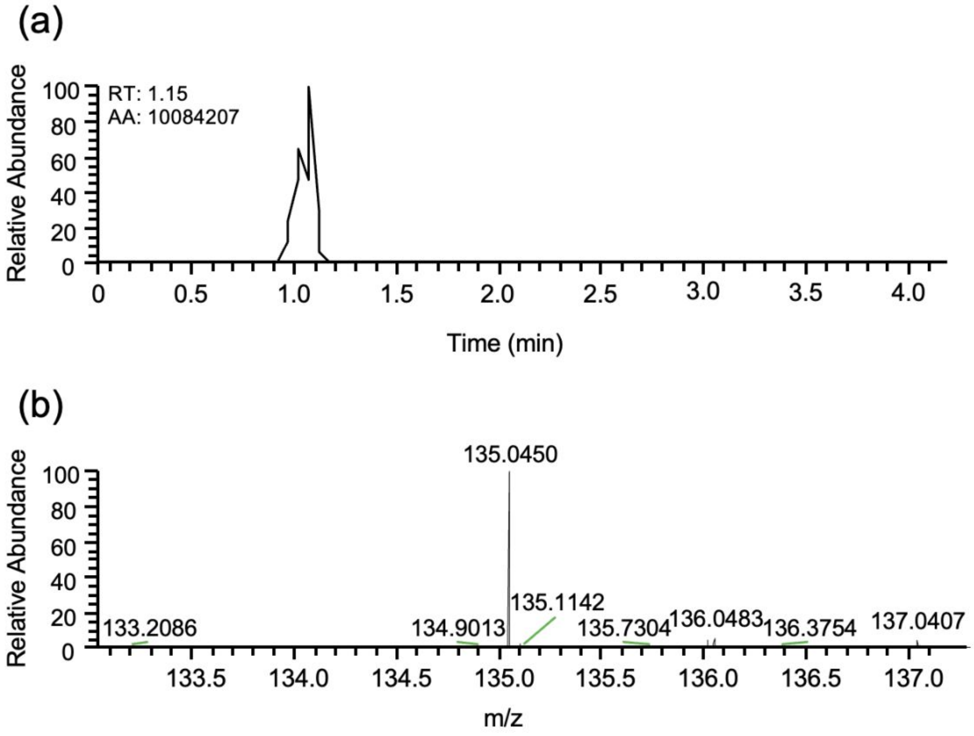

Figure S6. LC-MS chromatogram and mass spectrum of 12-day *E. huxleyi* culture extract. (a) LC-MS chromatogram in SIM mode at m/z = 135.045 and (b) EI mass spectrum covering the mass range from m/z 133 to 137.

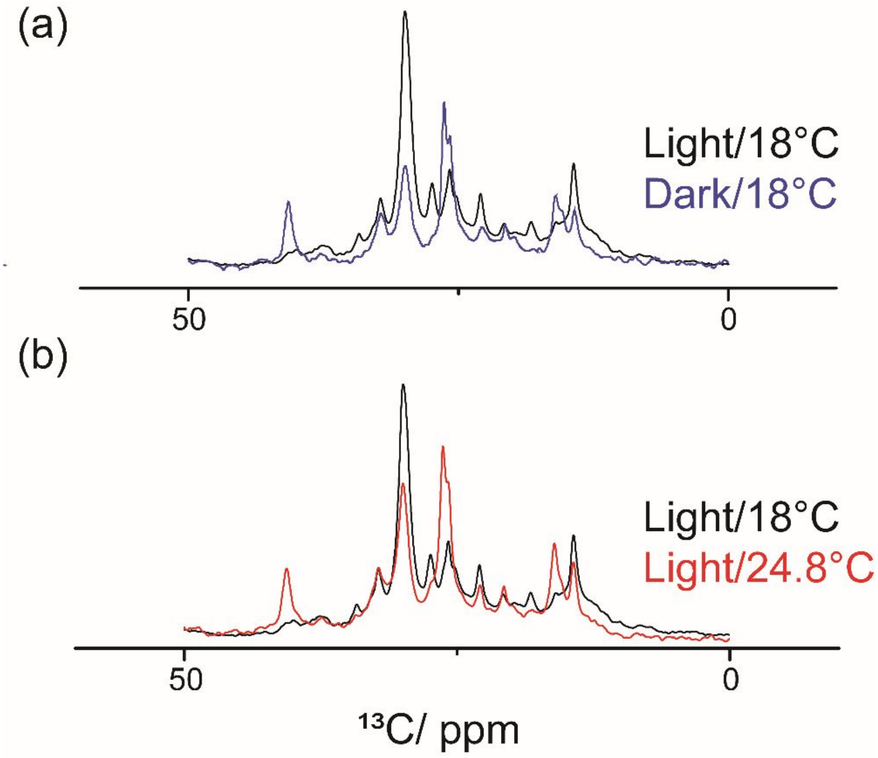

Figure S7. ^{13}C MAS spectra (0-50 ppm, aliphatic region) of coccolithophore pellets incubated for 24 h under different conditions following the addition of 50 µM [U-^{13}C] methionine. (a) Overlay of spectra at 18°C under light vs. darkness. (b) Overlay of spectra at 18°C vs. 24.8°C under light.
