# Supplementary material for: Investigation of Methionine Metabolism in Coccolithophore by In Situ Light-Coupled Nuclear Magnetic Resonance Spectroscopy
Source: J Phys Chem Lett. 2025 Jun 3;16(23):5800–5. doi: 10.1021/acs.jpclett.5c01316 (PMC12169660; doi:10.1021/acs.jpclett.5c01316)
Supplement: Supplementary file 2 [file jz5c01316_si_002.pdf]

jz-2025-01316h.R1

Name: Peer Review Information for "Investigation of methionine metabolism in coccolithophore by in situ light-coupled NMR spectroscopy"

First Round of Reviewer Comments

Reviewer: 1

Comments to the Author

This paper presents a novel biophysical approach to studying the metabolism of phytoplankton by a developing a light-coupled NMR method, and moreover a combination of solution-state and solid-state NMR spectroscopy. This method is very different and highly complementary of the established mass spectrometry methods which in this case encounter problems with stability of one of the studied metabolites. (which, one would expect, is a common problem with such studies) . The other advantage of the method is that it allows facile kinetic measurements under various environmental conditions and changes thereof.

This new biophysical method is applied to study the minute details of a process of tremendous importance, namely the oceanic sulfur cycle, and yields fine grain new details of the metabolism of methionine in coccolithophores.

I recommend publication of this very elegant work, with only one suggestion, and that is to provide a brief statement regarding the types of other studies that have employed light-coupled NMR.

Reviewer: 2

Comments to the Author

This is, in principle, a very nice study which uses in situ detection to monitor conversion of 13C-labelled Methionine to various metabolic products, depending on the environmental conditions. However, one major omission makes it difficult at this stage to assess the significance of the outcomes measured, as Authors did not provide details on what equipment they used for NMR detection, which spectrometers, fields, proheads, what was the acquisition time of the spectra shown on different figures. The concentrations that were measured and reported start from one micromolar, and often range around few micromols - not many NMR spectrometers and probeheads allow reliable measurement at such low concentrations. What NMR equipment the Authors used (liquid and solid-state), what field? What were the acquisition times and acquisition parameters?

Few other comments:

- At the beginning the effect of DMSP and DMS effect on climate and cloud forming is mentioned. Please clarify for the readers, whether these effects on climate are perceived to be positive or negative. Presumably calcification leads to positive effect of fixing the carbon, but what about DMS and DMSP, do they lead to heating or cooling of the planet?

- The authors mentioned cells settling down to the bottom of the tube in their liquid-state experiments. In the experiment with the in situ illumination, if the cells are at the bottom for the duration of the experiment, then presumably anything that they secrete to the media would also be largely confined to the bottom of the sample, outside or at the edge of NMR-active volume? With limited convection and mixing inside the liquid sample, the molecules may not diffuse far enough to be detected in the bulk of the NMR active area. This may significantly reduce the apparent concentration of the secreted compounds detected in the media, and sensitivity of the experiments. The Authors should comment on how they handled this issue and ensured sufficient sample mixing. Maybe the slow conversion kinetics observed in solution was due to the cells being confined to the very bottom, with very limited opportunity to exchange metabolites with the bulk of the solution?

- In few places the text appears to have extra % sign inserted between the numbers and the units, which may be typos or consequences of PDF conversion and some issues with the font conversions - needs to be fixed. E.g., shows as 58%mM, line 55 p11.

Author's Response to Peer Review Comments:

Response letter for "*Investigation of methionine metabolism in coccolithophore by in situ lightcoupled NMR spectroscopy*" Manuscript number: jz-2025-01316h Dear Editor:

We would like to thank the two reviewers for their valuable suggestions and critical comments and thank you for allowing us to address these concerns regarding our manuscript. Below, we would like to address each of the reviewer's comments point-by-point.

Reviewer: 1

Recommendation: This paper is publishable subject to minor revisions noted. Further review is not needed. Comments:

This paper presents a novel biophysical approach to studying the metabolism of phytoplankton by a developing a light-coupled NMR method, and moreover a combination of solution-state and solid-state NMR spectroscopy. This method is very different and highly complementary of the established mass spectrometry methods which in this case encounter problems with stability of one of the studied metabolites. (which, one would expect, is a common problem with such studies). The other advantage of the method is that it allows facile kinetic measurements under various environmental conditions and changes thereof. This new biophysical method is applied to study the minute details of a process of tremendous importance, namely the oceanic sulfur cycle, and yields fine grain new details of the metabolism of methionine in coccolithophores.

Response:
We sincerely appreciate the reviewer's recognition of the value of our work.

I recommend publication of this very elegant work, with only one suggestion, and that is to provide a brief statement regarding the types of other studies that have employed lightcoupled NMR.

Response:
We would like to thank the reviewer's suggestion to include a brief statement regarding other studies that have employed light-coupled NMR and cite the relevant references.

Revision made: The following statement was added at the beginning of the paragraph on page 6 and the relevant references, references 20 to 27, have been included in the revised manuscript. "Light-coupled NMR spectroscopy has been successfully applied to investigate photochemical processes and light-sensitive biomolecules.[20-27]."

Additional Questions: Urgency: High/ Significance: High/ Novelty: Top 10% / Scholarly Presentation: Top 10%/ Is the paper likely to interest a substantial number of physical chemists, not just specialists working in the authors' area of research?: Yes

Reviewer: 2

Recommendation: This paper is probably publishable, but major revision is needed; I do not need to see future revisions.

Comments:

This is, in principle, a very nice study which uses in situ detection to monitor conversion of 13Clabelled Methionine to various metabolic products, depending on the environmental conditions. **However, one major omission makes it difficult at this stage to assess the significance of the outcomes measured, as Authors did not provide details on what equipment they used for NMR detection, which spectrometers, fields, proheads, what was the acquisition time of the spectra shown on different figures.** The concentrations that were measured and reported start from one micromolar, and often range around few micromols - not many NMR spectrometers and probeheads allow reliable measurement at such low concentrations. What NMR equipment the Authors used (liquid and solid-state), what field? What were the acquisition times and acquisition parameters?

Response:
We apologize for omitting the important experimental parameters, which should have been documented in the manuscript. In the revised manuscript, all the important parameters were included as shown below.

Revision made:
Information regarding the spectrometer and probe is added in the paragraph on page 6, as described below.

“A Bruker NEO 850 MHz spectrometer, equipped with a 5mm TCI ($^1H/^{13}C/^{15}N$) CryoProbe with z-axis gradient, was employed in all the light-coupled NMR experiments.”

Details of *in situ* 2D[^{13}C,^1H] HSQC spectra are added in the paragraph on page 7, as shown below.

“All *in situ* 2D [^{13}C,^1H] HSQC spectra were acquired using the standard Bruker pulse sequence hsqcetgpsisp2.2, with the spectral widths set to 10.138 ppm and 140 ppm for the direct (^1H) dimension and indirect (^{13}C) dimension, respectively. Each spectrum was acquired with 512 complex points in the direct dimension and 50 complex points in the indirect dimension. The number of scans for each experiment was set to be 136. Due to the high salt content in the coccolithophore culture medium, the ^1H 90° pulse width at a power level of –12 dB was determined to be 14 μs.”

Details of 2D[^{13}C,^1H] HSQC spectra of standard compounds, involved in the methionine metabolism pathway, are added in the figure caption of Figure S3, as shown below.

“The 2D [^{13}C,^1H] HSQC spectra of the standard compounds, involved in the methionine metabolism pathway, dissolved in K/2 medium at a concentration of 1.5 mM. Each spectrum was acquired using the standard Bruker pulse sequence hsqcetgpsisp.2, with the spectral widths set to 14 ppm and 200 ppm for the direct (^1H) dimension and indirect (^{13}C) dimension, respectively. Each spectrum was acquired with 512 complex points in the direct dimension and 64 complex points in the indirect dimension. The number of scans for each experiment was set to be 12. Due to the high salt content in the K/2 medium, the ^1H 90° pulse width at a power level of –11.87 dB was determined to be 17 μs.”

Details of ^{13}C MAS NMR spectra are added on page 11, as shown below.

“All ^{13}C MAS NMR spectra were acquired on a Bruker wide-bore 11.7-T Avance III 500 MHz spectrometer equipped with a 3.2 mm triple-resonance magic angle spinning (MAS) probe. The sample spinning rate was set to 8 kHz, and the recycle delay was set to 5 s. Hahn echo spectra were recorded with ^{13}C radiofrequency pulses at a field strength of 50 kHz. The spectral width was 795 ppm, with a transmitter offset of -150.996 ppm. Final spectra were obtained by accumulating 10,000 scans.”

Few other comments:

1. At the beginning the effect of DMSP and DMS effect on climate and cloud forming is mentioned. Please clarify for the readers, whether these effects on climate are perceived to be positive or negative. **Presumably calcification leads to positive effect of fixing the carbon, but what about DMS and DMSP, do they lead to heating or cooling of the planet?**

Response:

We thank the reviewer for raising the important question regarding whether DMSP and its derivative DMS contribute to planetary warming or cooling. This is indeed a complex issue, as the oxidation products of DMS play a significant role in cloud and aerosol formation. These aerosols affect the Earth's heat balance through aerosol–radiation interactions, including the scattering and absorption of solar radiation. However, the net climatic effect of these processes remains uncertain and has not been confidently quantified in the literature. To address this, we have revised the manuscript to better reflect the complexity of DMS's influence on global temperatures.

Revision made:

The following description is added in the end of page 3 to describe the complexity of DMS's influence on global temperatures.

"DMS is crucial for climate regulation by forming cloud condensation nuclei (CCN) via its oxidation products such as dimethyl sulfoxide (DMSO) and sulfuric acid (H_2SO_4). These compounds contribute to cloud and aerosol formation, which in turn influence the Earth's heat balance by altering aerosol–radiation interactions via scattering and absorbing solar radiation. As a result, DMS emissions can ultimately impact global temperatures.[11-13]"

- The authors mentioned cells settling down to the bottom of the tube in their liquid-state experiments. **In the experiment with the in situ illumination, if the cells are at the bottom for the duration of the experiment, then presumably anything that they secrete to the media would also be largely confined to the bottom of the sample, outside or at the edge of NMRactive volume?** With limited convection and mixing inside the liquid sample, the molecules may not diffuse far enough to be detected in the bulk of the NMR active area. This may significantly reduce the apparent concentration of the secreted compounds detected in the media, and sensitivity of the experiments. The Authors should comment on how they handled this issue and ensured sufficient sample mixing. Maybe the slow conversion kinetics observed in solution was due to the cells being confined to the very

bottom, with very limited opportunity to exchange metabolites with the bulk of the solution?

Response:

We thank the reviewer for raising the important question regarding whether the cell secreted compounds in the NMR detection zone. The acquisition time for each spectrum was 6 h, which provided sufficient spectral sensitivity and, more importantly, allowed the secreted compounds to diffuse into the detection zone. Additionally, temperature regulation in the NMR probe was maintained through cooling air from the bottom, while light illumination was provided from the top. This setup inevitably created a temperature gradient, which led to sample mixing via convection.

Revision made:

We included an explanation in page 7 and an additional figure, Figure S2, in the supporting information to show that spectrum of the coccolithophore culture recorded immediately after thorough mixing was nearly identical to the one recorded before mixing.

“It is worth noting that the acquisition time for each spectrum was 6 h, which provided sufficient spectral sensitivity and, more importantly, allowed the secreted compounds to diffuse into the detection zone. As shown in Figure S2, the spectrum of the coccolithophore culture recorded immediately after thorough mixing is nearly identical to the one recorded before mixing.”

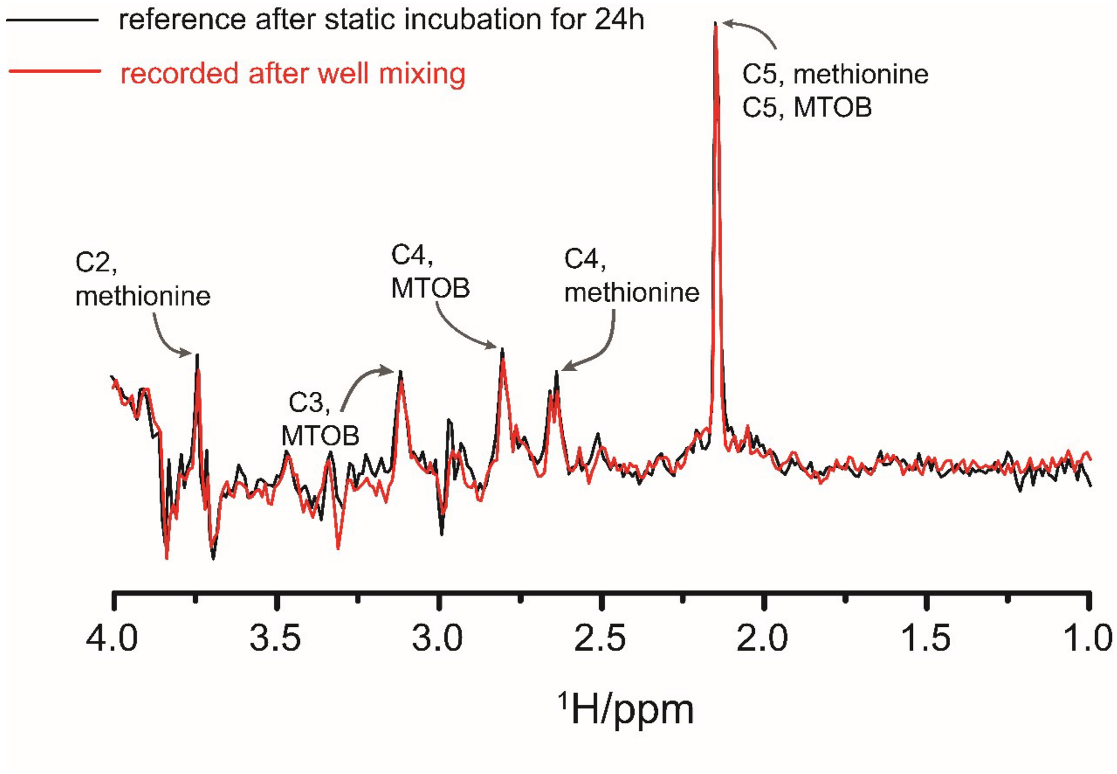

Figure S2. Overlay of 1D [^{13}C,^{1}H] HSQC spectra recorded from a coccolithophore sample after 24 h of static incubation and immediately after thorough mixing to ensure sample homogeneity.

The coccolithophore culture was grown to a density of 2 × 10^6 cells per mL and supplemented with

0.5 µL of 37 mM [U-^{13}C] labeled methionine and 37 µL of D_2O prior to static incubation at 18°C for 24 h. After recording the reference 1D HSQC spectrum, the sample was immediately mixed thoroughly, and a second spectrum was recorded. The experiments were performed on a Bruker AVIII 600 MHz spectrometer, equipped with a 5 mm TXI (^{1}H/^{13}C/^{15}N) CryoProbe with a z-axis gradient. The 1D [^{13}C,^{1}H] HSQC spectra were acquired using the standard Bruker pulse sequence hsqcetgpsisp2.2, with a spectral width of 10 ppm for the direct (^{1}H) dimension. The spectrum was acquired with 1024 complex points in the direct dimension, and 800 scans were collected. The ^{1}H 90° pulse width at a power level of -10.17 dB was determined to be 15.26 µs.

- In few places the text appears to have extra % sign inserted between the numbers and the units, which may be typos or consequences of PDF conversion and some issues with the font conversions - needs to be fixed. E.g., shows as 58%mM, line 55 p11.

Response:
We have carefully gone through the manuscript and noticed that these % symbols were not included in our word document.

Additional Questions: Urgency: High/ Significance: High/ Novelty: High/ Scholarly

Presentation: Moderate / Is the paper likely to interest a substantial number of physical chemists, not just specialists working in the authors' area of research?: Yes

Manuscript formatting request from editorial office

1. **Please include author names, article titles, journal name, publication year, and at least the first page number for the following incomplete journal references: 5, 14.**
Response & Revision:
We have updated references 5 and 14 to ensure that all citation details are complete as shown below.

(5) Broecker, W.; Clark, E. Ratio of coccolith $CaCO_3$ to foraminifera $CaCO_3$ in late Holocene deep sea sediments. *Paleoceanography* **2009**, *24* (3), PA3210.

(14) Gregory, G. J. , Boas, K. E. , and Boyd, E. F. The organosulfur compound dimethylsulfoniopropionate (DMSP) is utilized as an osmoprotectant by vibrio species. *Applied and Environmental Microbiology* **2020**, 87, e02235-02220.

Please include the URL and date of access for the following incomplete Website

reference(s): 27, 28.

Response & Revision:
Reference 27 and 28 are now reference 35 and 36 in the revised manuscript. We have updated the URL and date of access for references 35and 36 as shown below.

(35) National Oceanic and Admospheric Administration. *The Ongoing Marine Heat Waves in U.S. Waters, Explained*; 2023. https://www.noaa.gov/news/ongoing-marine-heat-waves-in-us-watersexplained (accessed 2023-08-24).

(36) Copernicus Climate Change Service. *Global Sea Surface Temperature Reaches a Record High*; 2023. https://climate.copernicus.eu/global-sea-surface-temperature-reaches-record-high (accessed 2024-11-24).

2. **Since your manuscript mentions different parts of your graphic, such as A and B, then the graphic/caption must clearly contain all mentioned parts.**

Response & Revision:

We have revised the figure captions of Figure 5, Figure S2 and Figure S3 to mention all relevant parts. The revisions were made as shown below.

Figure 5. 2D [^{13}C,^{1}H] HSQC spectra of coccolithophores grown in culture media at pH values of 8.18 (a), 8.0 (b), and 7.6 (c). The spectra were recorded 24 h after the addition of [U-^{13}C] labeled methionine.

Figure S3. The 2D [^{13}C,^{1}H] HSQC spectra of the standard compounds, involved in the methionine metabolism pathway including methionine (a), MTOB (b), MTHB (c), and DMSP (d). Each compound was dissolved in modified K/2 medium at a concentration of 1.5 mM. Each spectrum was acquired using the standard Bruker pulse sequence hsqcetgpsisp.2, with the spectral widths set to 14.0001 ppm and 200 ppm for the direct (^{1}H) dimension and indirect (^{13}C) dimension, respectively. Each spectrum was acquired with 512 complex points in the direct dimension and 64 complex points in the indirect dimension. The number of scans for each experiment was set to be

12. Due to the high salt content in the K/2 medium, the ^{1}H 90° pulse width at a power level of –

11.87 dB was determined to be 17 μs.

Figure S4. Flow cytometry dot plot and histogram of red fluorescence emitted by chlorophyll a at an emission wavelength of 690 nm for coccolithophore cultures grown to saturation under continuous illumination at 18 °C (a), and after 24 h of incubation with 50 μM [U-^{13}C] methionine under the following conditions: 18 °C with light (b), 18 °C in darkness (c), and 24.8 °C with light (d).

3. The TOC graphic should fit in an area no larger than 3.25 in. × 1.75 in. (approx. 8.25 cm × 4.45 cm) and should have adequate resolution and clarity. Confirm that all text is legible at this size.

Response & Revision:

We have prepared a new Table of Contents (TOC) graphic that meets the required criteria.

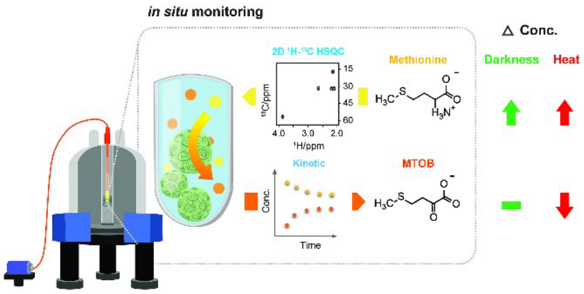

4. Your Supporting Information for Publication file should include a manuscript title, list of authors and their affiliations that matches exactly the title, author list, and affiliations in the manuscript file.

Response & Revision:

We have revised the title page of the supporting information so that all the information matches with the manuscript file.
